# Association Between Race and Comorbid Conditions Among Older Adults with Dementia

**DOI:** 10.3390/jcm13216368

**Published:** 2024-10-24

**Authors:** Parham Habibzadeh, Jennifer Albrecht

**Affiliations:** 1 Department of Medicine, University of Pittsburgh Medical Center, Montefiore Hospital, 200 Lothrop Street, N715, Pittsburgh, PA 15213, USA; 2 Department of Epidemiology and Public Health, School of Medicine, University of Maryland, Baltimore, MD 21201, USA; jalbrecht@som.umaryland.edu

**Keywords:** dementia, older adults, comorbidity, ethnic and racial minorities

## Abstract

**Background/Objective:** Dementia is estimated to affect over 150 million individuals by 2050. Individuals with dementia commonly suffer from other comorbid conditions which can affect quality of life and result in increased health care expenditures. We conducted this study to determine the frequency of comorbid conditions between representative samples of non-Hispanic Black and White US adults aged ≥65 with dementia. **Methods:** This cross-sectional study was conducted on non-Hispanic Black and White adults aged 65 and older with dementia whose data were retrieved from the National Hospital Ambulatory Medical Care Survey, 2016–2021, and the National Ambulatory Medical Care Survey, 2016, 2018, and 2019. Dementia was defined based on medical record abstraction. The exposure was Black vs. White race. The outcome was a sum of 13 comorbid conditions, including obesity, hypertension, cancer, cerebrovascular disease, congestive heart failure, and coronary artery disease, assessed in older adults with dementia. **Results:** A total of 1354 non-Hispanic (1175 White and 179 Black) participants were studied. The mean number of comorbid conditions, as well as the prevalence of obesity, cerebrovascular disease, congestive heart failure, and coronary artery disease, was significantly (*p*  <  0.01) higher in the Black vs. White study participants. The Black participants were more likely to have more than two comorbid conditions relative to those who were White (odds ratio 2.5; 95% confidence interval 1.6 to 3.7). **Conclusions:** A higher burden of comorbid conditions was observed among non-Hispanic Blacks compared to non-Hispanic White older adults with dementia. Future studies should examine the quality of life and health care utilization implications of this finding.

## 1. Introduction

Dementia is a global health issue that is estimated to affect more than 150 million individuals by 2050 [1]. As of 2020, one in every nine adults in the United States aged 65 and older was living with Alzheimer’s dementia; this number is projected to increase in the upcoming years due to the aging population [2]. Dementia imposes a significant financial burden on the health care system, with the average total cost per decedent with dementia exceeding those who had died of either cancer or heart disease by more than USD 100,000 [3]. It was estimated that the total monetary cost attributable to dementia in the United States was between USD 159 billion and 215 billion in 2010; this number is expected to increase by about 80% by 2040 [4]. In 2019, the economic burden of dementia worldwide amounted to USD 1.3 trillion [5]. Notably, out-of-pocket spending for patients with dementia is significantly higher than that in those without dementia, making it a significant financial burden to the patients and their caregivers [3,6]. For example, an investigation based on the Aging, Demographics, and Memory Study found that compared to those with normal cognition, people with dementia have more than three times the out-of-pocket spending for health care [6].

Comorbidities, which refer to the presence of more than one disease condition in the same individual, can complicate disease management due to sometimes conflicting treatments, medications, and monitoring measures. This complexity not only affects the quality of life, but also results in significantly increased health care expenditures. A study examining health expenditure associated with chronic multiple comorbidities revealed that about 60% of disease combinations had super-additive spending, which means that the total cost of treating several comorbid conditions is greater than the sum of treating each condition separately [7]. The results of a systematic review and meta-analysis on the costs associated with multiple comorbidities also support these findings and highlight the financial burden imposed on health care systems caused by the management of patients with multiple comorbid conditions [8].

Multimorbidity is common among individuals living with dementia, who often suffer from other comorbid conditions including hypertension, diabetes, and heart failure [9]. A study of 3013 older adults with dementia attending primary care clinics reported that they have 2.4 chronic conditions on average [10]. A study conducted on 400 individuals with a mean age of 79 years revealed that after adjusting for sex, age, blood pressure, and the use of antihypertensive medications, those with dementia are twice as likely as people without dementia to have a high left ventricular mass index, an independent predictor of cardiovascular events and organ damage in those with hypertension [11]. In a recent scoping review, Bergman et al. found that chronic comorbid conditions are not only more prevalent among people with dementia compared with those without dementia, but also affect the clinical presentation of dementia and influence the management of the patients and their quality of life [12]. The authors concluded that primary care providers should be aware of these circumstances and consider the management of patients with dementia in the presence of comorbidities.

Comorbid conditions have not only been shown to negatively affect the quality of life and well-being of those living with dementia, but are also the most important predictor of health care expenditures [13,14]. Health care expenditures have been estimated to be 10 times higher in those with greater than three comorbid health conditions compared to those without comorbidities [13]. Individuals with dementia with a higher number of comorbid conditions experience a higher number of hospital admissions, primary care consultations, and prescriptions [15,16]. A historical cohort study conducted on almost 5000 people diagnosed with dementia revealed that 92% of the studied people had one or more comorbid conditions. Compared with people with two or three comorbid conditions, those with six or more comorbidities had a 31% increase in the rate of primary care consultations; a 68% increase in prescriptions; a 62% increase in hospitalization; and a 56% increase in mortality [15]. Hospitalization can be harmful for those with dementia because they are more likely to be administered potentially inappropriate medications, are at a higher risk of delirium compared with individuals without dementia, and are less likely to be given adequate doses of painkillers [17,18,19].

Although racial disparities in comorbid conditions such as hypertension [20], diabetes [21], heart failure [22], and cancer [23] have been investigated in the general population, few studies have examined racial disparities in the frequency of comorbid conditions among older adults with dementia. However, given the aging population and the alarming increase in the number of individuals with dementia, it is vital to determine the racial disparities in comorbid conditions in this population. In addition, given the complex pathophysiology of aging, the magnitude of this disparity could vary among different age groups. Comorbid burden could be an important contributing factor to disparities in health care expenditures and the delivery of optimal care to this growing patient population. The objective of this study was to determine the association between race and the frequency of comorbid conditions among a nationally representative sample of older adults with dementia in the United States.

## 2. Methods

### 2.1. Study Design

We conducted a cross-sectional analysis of a nationally representative sample of patient visits in the United States based on the compiled data from two nationally representative databases—the National Hospital Ambulatory Medical Care Survey (NHAMCS) and the National Ambulatory Medical Care Survey (NAMCS)—from 2016 to 2021.

The sampling unit in NHAMCS was patient visits randomly collected from the Emergency Department (ED) visits across the 50 states and the District of Columbia based on a multi-stage probability design. Veterans Health Administration facilities, federal military facilities, and prisons were excluded. Geographically based primary sampling units, hospitals within these units, EDs within the hospitals, and patient visits to these EDs constituted the four-level probability sampling.

Data for the NAMCS were available for 2016, 2018, and 2019. NAMCS has implemented a three-stage probability sampling consisting of primary sampling units composed of geographic regions, practicing physicians within primary sampling units, and patient visits within the sample physicians. Only visits to non-federally employed office-based physician practices were included in this study. Physicians practicing in radiology, pathology, and anesthesiology specialties were excluded from this study.

In both surveys, visit-level data have been extracted by trained Census abstractors who have collected the relevant data by the chart review abstraction process. The medical diagnoses were established if the abstractors found any evidence of those conditions in the medical record or the treating physicians’ documentation. The data from the two surveys were combined to provide a better representation of the general population.

### 2.2. Study Population

The study population comprised older adults (aged ≥65 years) with a diagnosis of Alzheimer’s disease/dementia based on medical record abstraction.

### 2.3. Exposure

Patients’ race and ethnicity, the exposure, were identified based on abstracted information from the medical records. We restricted the study population to non-Hispanic (NH) Black and NH White individuals. Due to the small sample sizes within other race/ethnic categories, individuals from those ethnic/racial groups were excluded from the study.

### 2.4. Outcome

The presence of the following 13 comorbid conditions that are part of the Elixhauser comorbidity index [24] was assessed: ethanol or substance abuse, asthma, cancer, cerebrovascular disease (CVD), chronic kidney disease, chronic obstructive pulmonary disease (COPD), congestive heart failure (CHF), coronary artery disease, depression, diabetes mellitus (type I, type II, or non-specified), human immunodeficiency virus (HIV) infection, hypertension, and obesity. The total number of comorbid conditions for each patient was calculated by assigning one point for the presence of each of the specified conditions, thereby creating a cumulative index of comorbid condition burden (ranging from 0 to 13). The variable was then transformed into a binary outcome of high (>2) and low (≤2) comorbid conditions. The cut-off of 2 comorbid conditions was chosen based on the median value of the total number of comorbid conditions.

### 2.5. Statistical Analysis

SAS ver. 9.4 (SAS Institute, Cary, NC, USA) was used for data analysis. Survey weights were used in the analysis to produce national-level estimates and confidence intervals, but the raw frequencies are provided. The distribution of demographics and the medical conditions was assessed by race; the differences in their distributions were tested for statistical significance using the χ^2^ test for categorical variables, and linear regression analysis for continuous variables, implemented using SAS survey procedures. A logistic regression analysis was used to assess the crude association between the dichotomous comorbid condition burden (dependent variable) and race/ethnicity (independent variable). The variables that were associated with race at a *p*-value of < 0.1 were included in the final adjusted model. Odds ratios (ORs) along with their 95% confidence intervals (CIs) were reported.

## 3. Results

We studied 1354 NH (1175 White and 179 Black) patients with dementia aged ≥65 years. The proportion of females was significantly (*p* = 0.002) higher in NH Blacks (71.2%) compared to NH Whites (66.8%). There were no significant differences between the two race/ethnicity groups in terms of mean age, insurance status, setting (NHAMCS vs. NAMCS), and the study year (Table 1). The mean number of comorbid conditions, as well as the prevalence of obesity (86.1% NH Black, 68.7% NH White; *p* < 0.001), CVD (23.0% NH Black, 12.1% NH White; *p* = 0.01), CHF (23.6% NH Black, 8.8% NH White; *p* < 0.001), and coronary artery disease (23.6% NH Black, 8.8% NH White; *p* < 0.001), was significantly higher in NH Blacks than Whites. The prevalence of cancer was significantly (*p* = 0.01) lower among NH Blacks (5.3%) compared with Whites (11.3%).

NH Blacks had a higher mean number of comorbid conditions (2.8 vs. 2.2, *p* = 0.001) relative to NH Whites (Table 1).

Unadjusted logistic regression analysis revealed that the NH Black individuals were more likely to have >2 comorbid conditions relative to NH Whites (OR 2.5; 95% CI 1.6 to 3.7) (Table 2). The final model included a term for sex but resulted in no change to the effect estimate (i.e., no confounding).

## 4. Discussion

The presence of comorbid states in older adults with dementia poses a daunting challenge to health care professionals because of the complex interplay between the effects of multiple chronic disease conditions and the cognitive decline characteristic of dementia. The prevalence of comorbidities in the population of older adults with dementia significantly affects clinical outcomes, complicates strategies used for their management, and requires a multidisciplinary approach to care for this highly vulnerable patient population. The presence of comorbidities is associated with poor patient outcomes, but the signs and symptoms of dementia may cause them to be overlooked [25]. The presence of signs and symptoms of dementia can frequently result in the overshadowing of the existing comorbid conditions due to the cognitive and behavioral manifestations of dementia, which can potentially mask other medical issues. In certain instances, clinicians may erroneously attribute the observed signs and symptoms such as fatigue, weight loss, or sleep disturbances, to dementia itself, rather than recognizing the necessity for further inquiry into the potential indications of comorbid conditions. This can result in an underdiagnosis of comorbidities, as the focus on dementia’s prominent clinical features leads to a neglect of other underlying conditions. Furthermore, the complexity of dementia would make it challenging to differentiate symptoms caused by comorbidities from those directly related to cognitive decline. This can result in important comorbid conditions remaining undiagnosed and untreated, which can impact the patient’s overall health, outcome, and quality of life. It is, therefore, of paramount importance that health care providers maintain a high index of suspicion for comorbidities in patients with dementia to ensure providing comprehensive care.

We found that in a nationally representative sample of older adults with dementia, NH Blacks were more than twice as likely to have more than two comorbid conditions. In a study on 107 patients with a confirmed diagnosis of dementia attending primary care practice health centers, Schubert et al. reported that these patients had 2.4 chronic conditions on average [10]. This is consistent with our findings (2.8 among NH Blacks and 2.2 among NH Whites). Obesity, coronary artery diseases, CVD, and CHF were more prevalent among older NH Blacks with dementia compared with their NH White counterparts. This is consistent with findings from a registry-based study conducted in Finland which found that cardiovascular and cerebrovascular diseases and metabolic disorders were common patterns of comorbidity observed in the last years of life of older adults with dementia [26].

In our series of older NH people with dementia, we observed a higher prevalence of comorbid conditions in Blacks compared with Whites. The disparity in the prevalence of comorbid states between NH Blacks and NH Whites has significant implications, such as an increased likelihood of hospitalization and ultimately greater health care expenditures in this population. In addition, the presence of multimorbidity leaves individuals who are Black more vulnerable to developing complications requiring hospitalization and resulting in higher health care costs [27].

We found that obesity was more prevalent among NH Blacks compared with NH Whites. Obesity is a well-established risk factor for CVD, coronary artery disease, and CHF. Obesity is also associated with hypertension, a risk factor for CVD; coronary artery disease; and CHF [28]. Finally, obesity is associated with dyslipidemia—increased low-density lipoprotein and triglyceride levels and decreased high-density lipoprotein levels—which accelerates atherosclerosis [29]. According to the Center for Disease Control and Prevention (CDC), the overall age-adjusted prevalence of obesity among adults aged 20 years and more in 2017–2018 was 49.6% in NH Blacks as compared with 42.2% in NH Whites [30]. The higher prevalence of obesity among NH Blacks can thus increase the risk of CVD and CHF. It has been shown that among patients with heart failure with preserved ejection fraction, those with obesity are at increased risk of heart failure-related hospitalizations [31]. Another recent study revealed that the risk of hospitalization in patients with CVD and severe comorbidities is more than double that in patients with CVD alone (44% vs. 20%) [32].

We found that compared with the older NH Black individuals with dementia, cancers were more prevalent in the older NH Whites with dementia, consistent with the findings of Zamrini, et al. [33]. Prior literature has demonstrated that between 2000 and 2010, the mortality from all-site malignancies in the United States was consistently higher in Black compared with White adults and survival time was shorter [23]. A recent cohort study on more than 274,000 NH Black and White women with uterine cancer also revealed that among patients aged 65 years and over, the adjusted risk of death in Black individuals is almost twice that in White patients [34]. Higher mortality rates among NH Black patients with cancer could possibly have contributed to the lower prevalence of cancer among the Black older adults with dementia observed in this study.

Other contributing factors include the underdiagnosis and delayed diagnosis of malignancies among NH Black patients. For instance, the screening rate for cervical cancer is 53% in NH Blacks as compared with 64% in NH Whites [35]. In a recent study of more than 26,000 women, it was found that mammography completion was 15–26% lower in Blacks as compared with Whites [36]. In a historical cohort study conducted on more than 1,700,000 participants aged 50–75 years from the United States, it was found that compared with NH Whites, the adjusted odds of completing colorectal cancer screening were 14% (95% CI, 12% to 16%) lower for NH Blacks in health care systems with high screening rates [37]. Another recent study on more than 2500 women from the United States who underwent breast imaging revealed that the waiting time for a tissue diagnosis in Black women with abnormal findings is 75% longer than that in White women with abnormal findings [38]. An analysis of Texas Cancer Registry data between 2005 and 2017 for patients aged 66 years and more (more than 85,000 records) reveals that after appropriately adjusting the model for confounding variables, NH Black women have 11% higher odds of a diagnosis of their breast cancer at a late stage compared with their White counterparts. For colorectal cancer, the odds of late-stage diagnosis were 29% higher for NH Blacks than for NH Whites [39]. A lower detection rate and the delayed diagnosis of malignant disorders lead to diagnosis at more advanced stages with unfavorable prognosis. While socioeconomic, demographic, and clinical differences may account for some of the above-mentioned disparities, some authors attribute the observed differences to systemic effects of racism against Black people, a factor that is arguably considered one of the major contributors to the differences in outcomes reported between Black and White patients with malignancies [40].

We found that the prevalence of hypertension, although higher in the Blacks compared with the Whites, was not significantly different between the two groups (Table 1). These were, in part, in keeping with the findings of the National Center for Health Statistics (NCHS) 2019 report that showed that the prevalence of obesity and hypertension was higher in NH Blacks compared with NH Whites from 1999 to 2017 [41]. Another study conducted on more than 50,000 adults revealed that systolic blood pressure was consistently higher in NH Blacks compared with NH Whites from 1999 to 2018 [20]. According to the CDC, the overall age-adjusted prevalence of hypertension among adults aged 18 years and over in 2017–2018 was 57.1% in NH Blacks as compared with 43.6% in NH Whites [42]. Of note, a study of Medicare beneficiaries found that hypertension disproportionately increased the rate of Alzheimer’s disease in Black individuals compared with White individuals [43]. The increased prevalence of obesity and hypertension, the two risk factors for CVD; coronary artery disease; and CHF results in an increased risk of these diseases, which is in agreement with the higher prevalence of CVD, coronary artery disease, and CHF observed among the studied NH Blacks compared with NH Whites.

Vascular dementia comprises a heterogeneous group of brain disorders. Vascular causes (micro- and macro-angiopathies) are among the most common etiologies of dementia. The incidence of vascular dementia varies considerably depending on the specific diagnostic criteria used [44,45]. An autopsy study based on the National Alzheimer’s Coordinating Center’s Neuropathology dataset on 110 Blacks and 2500 Whites showed that vascular dementia is twice as common in Blacks as compared with Whites [46]. We did not have information on subtypes of dementia in the current study, but a higher prevalence of CVD and coronary artery disease, which could be considered tantamount to atherosclerosis, in NH Blacks compared with NH Whites suggests a higher rate of vascular dementia among the Blacks in our study. A nationwide study on more than 50,000 US participants revealed a higher prevalence of CVD risk factors in NH Black adults compared with NH Whites [47]. However, the observed difference in the 10-year CAD risk was attenuated after adjusting for the social determinants of health [48]—socioeconomic status, neighborhood, and physical environment, social support networks, and access to health care services [47]. In the current study, NH Blacks were more likely than NH Whites to be insured through Medicaid (11.5% vs. 6.9%), a proxy for low income, but this association did not reach statistical significance.

Multilevel disparities in access to high-quality health care services, economic development, and public awareness that are already known to be major contributors to disparities in health conditions including diabetes and CVD are also likely to have played a major role in the higher burden of comorbid conditions observed among NH Black older adults with dementia compared with their White counterparts [49,50]. This highlights the importance of the implementation of public health measures and policies to address the unmet needs of this population.

A strength of the current study is that it uses two nationally representative databases (NAMCS and NHAMCS) to study the burden of comorbidity among older NH Black and NH White adults with dementia. However, our study was limited by the small sample sizes which prohibited the examination of other racial and ethnic groups, the lack of longitudinal data, and also the unavailability of important variables, such as dementia severity and subtype, the social determinants of health, and biological factors (e.g., genetic features).

## 5. Conclusions

In conclusion, NH Black older adults with dementia were more than twice as likely to have more than two comorbidities relative to their White counterparts. The significant disparity observed in the prevalence of comorbidities among the studied NH Black older adults with dementia, as compared with their White counterparts, underscores a critical public health concern. This finding would suggest a complex interplay of factors that contribute to the overall health burden in older US adults with dementia. Future research studies should be conducted to delve deeper into the ramifications of such disparities on the quality of life, which encompasses both physical and mental well-being, and health care expenditures, which are often a source of financial burden for patients, families, and the health care system. Taking into account the social determinants of health (e.g., education, and neighborhood-level poverty) along with biological factors (e.g., genetic features) may provide a more comprehensive understanding of the challenges faced by this vulnerable patient population. This holistic approach may ultimately reveal underlying mechanisms that contribute to the development and progression of dementia and its associated comorbidities. It is also imperative to assess the effectiveness of the currently used surveillance strategies for patients with dementia, and cardiovascular and cerebrovascular diseases in populations at an increased risk. By focusing on the social determinants of health, researchers may identify targeted interventions and implement them to appropriately address the specific needs of these communities. Such efforts could lead to the development of more equitable health care practices and policies, ultimately improving the outcomes for high-risk groups and fostering a more inclusive health care environment.

## Figures and Tables

**Table 1:** Baseline demographic and comorbid characteristics among the adults aged 65 and older with dementia identified in the NHAMCS (2016–2021) and NAMCS (2016, 2018, and 2019), by race. The frequencies and weighted percentages are presented.

Variable	Non-Hispanic White (*n* = 1175)	Non-Hispanic Black (*n* = 179)	*p*-Value *
Age, Mean (95% CI)	81.3 (80.4–82.2)	81.6 (81.1–82.2)	0.9
Sex, *n* (weighted%)			
Female	728 (66.8)	118 (71.2)	0.002
Male	447 (33.2)	61 (28.8)	
Insurance, *n* (weighted%)			
Medicare	922 (77.7)	98 (64.5)	0.2
Medicaid	131 (6.9)	50 (11.5)	
Private Insurance	40 (9.6)	11(19.2)	
Others	82 (5.8)	20 (4.9)	
Setting			
NHAMCS	1002 (33.6)	157 (33.1)	1
NAMCS	173 (66.4)	22 (66.9)	
Year			
2016	256 (22.7)	25 (13.2)	0.2
2017	162 (6.3)	17 (3.5)	
2018	225 (21.6)	39 (20.6)	
2019	246 (38.3)	40 (50.3)	
2020	145 (6.0)	27 (5.0)	
2021	141 (5.0)	31 (7.4)	
Comorbid Conditions			
ETOH or Substance Abuse	33 (1.5)	13 (2.9)	0.3
Asthma	49 (1.8)	11 (1.9)	0.9
Cancer	128 (11.3)	21 (5.3)	0.01
Cerebrovascular Disease	207 (12.1)	29 (23.0)	0.01
Chronic Kidney Disease	169 (14.3)	37 (27.3)	0.08
COPD	166 (14.4)	24 (11.2)	0.3
CHF	168 (8.8)	31 (23.6)	0.0005
Coronary Artery Disease	272 (18.2)	40 (40.6)	<0.0001
Depression	260 (27.6)	32 (26.0)	0.8
Diabetes Mellitus	280 (28.3)	62 (28.0)	1
Hypertension	399 (39.3)	64 (52.5)	0.2
Obesity	745 (68.7)	121 (86.1)	<0.0001
Mean Number of Comorbid Conditions (95% CI) ^†^	2.2 (2.0–2.4)	2.8 (2.7–2.9)	0.001

* *p*-values were calculated based on linear regression analysis for age and the sum of comorbid conditions; χ^2^ test, for others. Percentages represent the weighted frequencies. ^†^ Calculated based on the conditions listed in the Elixhauser criteria. Abbreviations: NHAMCS, National Hospital Ambulatory Medical Care Survey; NAMCS, National Ambulatory Medical Care Survey; COPD, chronic obstructive pulmonary disease; ETOH, ethanol; CHF, congestive heart failure.

**Table 2:** Association between race/ethnicity and comorbid condition burden among adults aged 65 and older with dementia from NHAMCS (2016–2021) and NAMCS (2016, 2018, and 2019) assessed using logistic regression analysis.

Model	Odds Ratio (95% CI)
**Unadjusted**	
Non-Hispanic Black	2.5 (1.6 to 3.7)
Non-Hispanic White	1 (Ref)
**Adjusted for sex**	
Non-Hispanic Black	2.5 (1.6 to 3.7)
Non-Hispanic White	1 (Ref)

## Data Availability

The datasets used in the current study are publicly available from the CDC National Center for Health Statistics website (https://www.cdc.gov/nchs/dhcs/index.htm, accessed on 25 August 2024).

## References

[1] GBD 2019 Dementia Forecasting Collaborators (2022). Estimation of the global prevalence of dementia in 2019 and forecasted prevalence in 2050: An analysis for the Global Burden of Disease Study 2019. Lancet Public Health.

[2] Rajan K.B., Weuve J., Barnes L.L., McAninch E.A., Wilson R.S., Evans D.A. (2021). Population estimate of people with clinical Alzheimer’s disease and mild cognitive impairment in the United States (2020–2060). Alzheimers Dement..

[3] Kelley A.S., McGarry K., Gorges R., Skinner J.S. (2015). The burden of health care costs for patients with dementia in the last 5 years of life. Ann. Intern. Med..

[4] Hurd M.D., Martorell P., Delavande A., Mullen K.J., Langa K.M. (2013). Monetary costs of dementia in the United States. N. Engl. J. Med..

[5] Wimo A., Seeher K., Cataldi R., Cyhlarova E., Dielemann J.L., Frisell O., Guerchet M., Jonsson L., Malaha A.K., Nichols E. (2023). The worldwide costs of dementia in 2019. Alzheimers Dement..

[6] Delavande A., Hurd M.D., Martorell P., Langa K.M. (2013). Dementia and out-of-pocket spending on health care services. Alzheimers Dement..

[7] Chang A.Y., Bryazka D., Dieleman J.L. (2023). Estimating health spending associated with chronic multimorbidity in 2018: An observational study among adults in the United States. PLoS Med..

[8] Tran P.B., Kazibwe J., Nikolaidis G.F., Linnosmaa I., Rijken M., van Olmen J. (2022). Costs of multimorbidity: A systematic review and meta-analyses. BMC Med..

[9] Poblador-Plou B., Calderon-Larranaga A., Marta-Moreno J., Hancco-Saavedra J., Sicras-Mainar A., Soljak M., Prados-Torres A. (2014). Comorbidity of dementia: A cross-sectional study of primary care older patients. BMC Psychiatry.

[10] Schubert C.C., Boustani M., Callahan C.M., Perkins A.J., Carney C.P., Fox C., Unverzagt F., Hui S., Hendrie H.C. (2006). Comorbidity profile of dementia patients in primary care: Are they sicker?. J. Am. Geriatr. Soc..

[11] Scuteri A., Coluccia R., Castello L., Nevola E., Brancati A.M., Volpe M. (2009). Left ventricular mass increase is associated with cognitive decline and dementia in the elderly independently of blood pressure. Eur. Heart J..

[12] Bergman H., Borson S., Jessen F., Krolak-Salmon P., Pirani A., Rasmussen J., Rodrigo J., Taddeo D. (2023). Dementia and comorbidities in primary care: A scoping review. BMC Prim. Care.

[13] Zhu C.W., Cosentino S., Ornstein K.A., Gu Y., Andrews H., Stern Y. (2017). Interactive Effects of Dementia Severity and Comorbidities on Medicare Expenditures. J. Alzheimers Dis..

[14] Sabatini S., Martyr A., Hunt A., Gamble L.D., Matthews F.E., Thom J.M., Jones R.W., Allan L., Knapp M., Victor C. (2024). Comorbid health conditions and their impact on social isolation, loneliness, quality of life, and well-being in people with dementia: Longitudinal findings from the IDEAL programme. BMC Geriatr..

[15] Browne J., Edwards D.A., Rhodes K.M., Brimicombe D.J., Payne R.A. (2017). Association of comorbidity and health service usage among patients with dementia in the UK: A population-based study. BMJ Open.

[16] Shepherd H., Livingston G., Chan J., Sommerlad A. (2019). Hospitalisation rates and predictors in people with dementia: A systematic review and meta-analysis. BMC Med..

[17] Lichtner V., Dowding D., Allcock N., Keady J., Sampson E.L., Briggs M., Corbett A., James K., Lasrado R., Swarbrick C. (2016). The assessment and management of pain in patients with dementia in hospital settings: A multi-case exploratory study from a decision making perspective. BMC Health Serv. Res..

[18] White N., Leurent B., Lord K., Scott S., Jones L., Sampson E.L. (2017). The management of behavioural and psychological symptoms of dementia in the acute general medical hospital: A longitudinal cohort study. Int. J. Geriatr. Psychiatry.

[19] Ryan D.J., O’Regan N.A., Caoimh R.O., Clare J., O’Connor M., Leonard M., McFarland J., Tighe S., O’Sullivan K., Trzepacz P.T. (2013). Delirium in an adult acute hospital population: Predictors, prevalence and detection. BMJ Open.

[20] Hardy S.T., Chen L., Cherrington A.L., Moise N., Jaeger B.C., Foti K., Sakhuja S., Wozniak G., Abdalla M., Muntner P. (2021). Racial and Ethnic Differences in Blood Pressure Among US Adults, 1999–2018. Hypertension.

[21] Cheng Y.J., Kanaya A.M., Araneta M.R.G., Saydah S.H., Kahn H.S., Gregg E.W., Fujimoto W.Y., Imperatore G. (2019). Prevalence of Diabetes by Race and Ethnicity in the United States, 2011–2016. JAMA.

[22] Glynn P., Lloyd-Jones D.M., Feinstein M.J., Carnethon M., Khan S.S. (2019). Disparities in Cardiovascular Mortality Related to Heart Failure in the United States. J. Am. Coll. Cardiol..

[23] O’Keefe E.B., Meltzer J.P., Bethea T.N. (2015). Health disparities and cancer: Racial disparities in cancer mortality in the United States, 2000–2010. Front. Public Health.

[24] Elixhauser A., Steiner C., Harris D.R., Coffey R.M. (1998). Comorbidity measures for use with administrative data. Med. Care.

[25] Subramaniam H. (2019). Co-morbidities in dementia: Time to focus more on assessing and managing co-morbidities. Age Ageing.

[26] Vargese S.S., Halonen P., Raitanen J., Forma L., Jylha M., Aaltonen M. (2021). Comorbidities in dementia during the last years of life: A register study of patterns and time differences in Finland. Aging Clin. Exp. Res..

[27] Mohamud M.A., Campbell D.J.T., Wick J., Leung A.A., Fabreau G.E., Tonelli M., Ronksley P.E. (2023). 20-year trends in multimorbidity by race/ethnicity among hospitalized patient populations in the United States. Int. J. Equity Health.

[28] Koskinas K.C., Van Craenenbroeck E.M., Antoniades C., Bluher M., Gorter T.M., Hanssen H., Marx N., McDonagh T.A., Mingrone G., Rosengren A. (2024). Obesity and cardiovascular disease: An ESC clinical consensus statement. Eur. Heart J..

[29] Dwivedi A.K., Dubey P., Cistola D.P., Reddy S.Y. (2020). Association Between Obesity and Cardiovascular Outcomes: Updated Evidence from Meta-analysis Studies. Curr. Cardiol. Rep..

[30] Hales C.M., Carroll M.D., Fryar C.D., Ogden C.L. Prevalence of Obesity and Severe Obesity Among Adults: United States, 2017–2018. https://www.cdc.gov/nchs/data/databriefs/db360-h.pdf.

[31] Morgen C.S., Haase C.L., Oral T.K., Schnecke V., Varbo A., Borlaug B.A. (2023). Obesity, Cardiorenal Comorbidities, and Risk of Hospitalization in Patients with Heart Failure with Preserved Ejection Fraction. Mayo Clin. Proc..

[32] Soysaler C.A., Andrei C.L., Ceban O., Sinescu C.J. (2023). Comorbidity Patterns in Patients at Cardiovascular Hospital Admission. Medicines.

[33] Zamrini E., Parrish J.A., Parsons D., Harrell L.E. (2004). Medical comorbidity in black and white patients with Alzheimer’s disease. South. Med. J..

[34] Kucera C.W., Tian C., Tarney C.M., Presti C., Jokajtys S., Winkler S.S., Casablanca Y., Bateman N.W., Mhawech-Fauceglia P., Wenzel L. (2023). Factors Associated with Survival Disparities Between Non-Hispanic Black and White Patients with Uterine Cancer. JAMA Netw. Open.

[35] Spencer J.C., Kim J.J., Tiro J.A., Feldman S.J., Kobrin S.C., Skinner C.S., Wang L., McCarthy A.M., Atlas S.J., Pruitt S.L. (2023). Racial and Ethnic Disparities in Cervical Cancer Screening from Three U.S. Healthcare Settings. Am. J. Prev. Med..

[36] Ganguly A.P., Baker K.K., Redman M.W., McClintock A.H., Yung R.L. (2023). Racial disparities in the screening mammography continuum within a heterogeneous health care system. Cancer.

[37] Burnett-Hartman A.N., Mehta S.J., Zheng Y., Ghai N.R., McLerran D.F., Chubak J., Quinn V.P., Skinner C.S., Corley D.A., Inadomi J.M. (2016). Racial/Ethnic Disparities in Colorectal Cancer Screening Across Healthcare Systems. Am. J. Prev. Med..

[38] Manik R., Grady C.B., Ginzberg S.P., Edmonds C.E., Conant E.F., Hubbard R.A., Fayanju O.M. (2024). Racial Disparities and Strategies for Improving Equity in Diagnostic Follow-Up for Abnormal Screening Mammograms. JCO Oncol. Pract..

[39] Nicot-Cartsonis M.S., Digbeu B.D.E., Raji M.A., Kuo Y.F. (2023). Disparities in Late-Stage Breast and Colorectal Cancer Diagnosis Among Hispanic, Non-Hispanic White, and Non-Hispanic Black Patients: A Retrospective Cohort Study of Texas Medicare Beneficiaries. J. Racial Ethn. Health Disparities.

[40] Simon M.S., Raychaudhuri S., Hamel L.M., Penner L.A., Schwartz K.L., Harper F.W.K., Thompson H.S., Booza J.C., Cote M., Schwartz A.G. (2021). A Review of Research on Disparities in the Care of Black and White Patients with Cancer in Detroit. Front. Oncol..

[41] National Center for Health Statistics (NCHS) Health, United States Spotlight: Racial and Ethnic Disparities in Heart Disease. https://www.cdc.gov/nchs/hus/spotlight/HeartDiseaseSpotlight_2019_0404.pdf.

[42] Ostchega Y., Fryar C.D., Nwankwo T., Nguyen D.T. Hypertension Prevalence Among Adults Aged 18 and Over: United States, 2017–2018. https://www.cdc.gov/nchs/data/databriefs/db364-h.pdf.

[43] Steenland K., Tan Y., Wingo T., Shi L., Xiao S., Wharton W. (2022). The effect of race and co-morbidities on Alzheimer’s disease based on Medicare data. Alzheimer’s Dement..

[44] Rockwood K., Wentzel C., Hachinski V., Hogan D.B., MacKnight C., McDowell I. (2000). Prevalence and outcomes of vascular cognitive impairment. Vascular Cognitive Impairment Investigators of the Canadian Study of Health and Aging. Neurology.

[45] De Reuck J., Deramecourt V., Cordonnier C., Pasquier F., Leys D., Maurage C.A., Bordet R. (2016). The incidence of post-mortem neurodegenerative and cerebrovascular pathology in mixed dementia. J. Neurol. Sci..

[46] Graff-Radford N.R., Besser L.M., Crook J.E., Kukull W.A., Dickson D.W. (2016). Neuropathologic differences by race from the National Alzheimer’s Coordinating Center. Alzheimers Dement..

[47] He J., Zhu Z., Bundy J.D., Dorans K.S., Chen J., Hamm L.L. (2021). Trends in Cardiovascular Risk Factors in US Adults by Race and Ethnicity and Socioeconomic Status, 1999–2018. JAMA.

[48] Havranek E.P., Mujahid M.S., Barr D.A., Blair I.V., Cohen M.S., Cruz-Flores S., Davey-Smith G., Dennison-Himmelfarb C.R., Lauer M.S., Lockwood D.W. (2015). Social Determinants of Risk and Outcomes for Cardiovascular Disease: A Scientific Statement From the American Heart Association. Circulation.

[49] Lopez-Neyman S.M., Davis K., Zohoori N., Broughton K.S., Moore C.E., Miketinas D. (2022). Racial disparities and prevalence of cardiovascular disease risk factors, cardiometabolic risk factors, and cardiovascular health metrics among US adults: NHANES 2011–2018. Sci. Rep..

[50] Hassan S., Gujral U.P., Quarells R.C., Rhodes E.C., Shah M.K., Obi J., Lee W.H., Shamambo L., Weber M.B., Narayan K.M.V. (2023). Disparities in diabetes prevalence and management by race and ethnicity in the USA: Defining a path forward. Lancet Diabetes Endocrinol..

[51] HIPAA Privacy Rule Questions & Answers for NAMCS. https://www.cdc.gov/nchs/namcs-health-center/about/privacy-and-confidentiality.html.

[52] HIPAA Privacy Rule Questions & Answers for NHAMCS. https://www.cdc.gov/nchs/namcs/about/?CDC_AAref_Val=https://www.cdc.gov/nchs/ahcd/nhamcs_hipaa_privacy.htm.

